# Role of home visits by volunteer community health workers: to improve the coverage of micronutrient powders in rural Bangladesh

**DOI:** 10.1017/S1368980020000038

**Published:** 2021-04

**Authors:** Haribondhu Sarma, Mduduzi NN Mbuya, Md Tariqujjaman, Mahfuzur Rahman, Sufia Askari, Rudaba Khondker, Sabiha Sultana, Shaima Arzuman Shahin, Thomas J Bossert, Cathy Banwell, Lynnette M Neufeld, Tahmeed Ahmed, Catherine D’Este

**Affiliations:** 1 Nutrition and Clinical Services Division, icddr,b, Dhaka 1212, Bangladesh; 2 Research School of Population Health, The Australian National University, Canberra, ACT 2601, Australia; 3 Global Alliance for Improved Nutrition, Dhaka 1212, Bangladesh; 4 Child Health and Development, The Children’s Investment Fund Foundation, London W1S 2FT, UK; 5 Health Nutrition and Population, BRAC, Dhaka 1212, Bangladesh; 6 Department of Global Health and Population, The Harvard T.H. Chan School of Public Health, Boston, MA 02115, USA

**Keywords:** Home visit, Volunteer community health workers, Home fortification, Micronutrient powder, Coverage

## Abstract

**Objective:**

We assessed the role of home visits by *Shasthya Shebika* (SS) – female volunteer community health workers (CHWs) – in improving the distribution of micronutrient powder (MNP), and explored the independent effects of caregiver–provider interaction on coverage variables.

**Design:**

We used data from three cross-sectional surveys undertaken at baseline (*n* 1927), midline (*n* 1924) and endline (*n* 1540) as part of an evaluation of a home fortification programme. We defined an exposure group as one that had at least one SS visit to the caregiver’s household in the 12 months preceding the survey considering three outcome variables – message (ever heard), contact (ever used) and effective coverage (regular used) of MNP. We performed multiple logistic regressions to explore the determinants of coverage, employed an ‘interaction term’ and calculated an odds ratio (OR) to assess the modifying effect of SS’s home visits on coverage.

**Settings:**

Sixty-eight sub-districts from ten districts of Bangladesh.

**Participants:**

Children aged 6–59 months and their caregivers.

**Results:**

A home visit from an SS positively impacts message coverage at both midline (ratio of OR 1·70; 95 % CI 1·25, 2·32; *P* < 0·01) and endline (ratio of OR 3·58; 95 % CI 2·22, 5·78; *P* < 0·001), and contact coverage both at midline (ratio of OR 1·48; 95 % CI 1·06, 2·07; *P* = 0·021) and endline (ratio of OR 1·74; 95 % CI 1·23, 2·47; *P* = 0·002). There was no significant effect of a SS’s home visit on effective coverage.

**Conclusions:**

The households visited by BRAC’s volunteer CHWs have better message and contact coverage among the children aged 6–59 months.

Fortifying domestic foods with micronutrient powder (MNP) is an efficacious and cost-effective intervention to address micronutrient deficiencies in those with the greatest potential to benefit^(1,2)^. The WHO recommends the use of home fortification with MNP for children aged 6–23 and 2–12 months to improve their iron status and anaemia in populations where anaemia is a public health problem^(3)^. Despite WHO recommendation and availability of this and other interventions, a high prevalence of anaemia and other micronutrient deficiencies persist in low-income settings^(4,5)^ such as Bangladesh. Among the many problems is poor coverage of interventions at the community level^(6,7)^. Improving the coverage (e.g. the proportion of population in a particular area receiving home fortification interventions) is critical to reduce the risk or prevalence of micronutrient deficiency at the population level.

There are many factors that hinder a successful implementation of a home fortification programme with MNP in low-income countries^(8)^. These include barriers at both the service delivery level and beneficiary level. The involvement of frontline workers (health workers, community health workers (CHWs), vendors and pharmacists) can improve access and acceptance^(9–12)^. Caregivers’ awareness, motivation and skill in applying MNP to children’s food may improve uptake and sustained use^(12)^. The latter requires regular communication between caregivers and community-based providers of MNP. Consequently, home fortification interventions implemented by CHWs have included regular home contact and counselling with caregivers.

## Maternal Infant and Young Child Nutrition programme in Bangladesh

The National Strategy for Prevention and Control of Anaemia in Bangladesh has recommended adding MNP to the diets of 6–59-month-old children^(13)^. Between 2014 and 2018, BRAC (the Bangladesh Rural Advancement Committee) scaled up its home fortification programme using MNP to a national level as part of its Maternal Infant and Young Child Nutrition (MIYCN) programme. The programme aimed to reduce anaemia by 10 % among 6–59-month-old children by increasing the coverage of MNP. It took a community-based approach using BRAC’s CHWs to sell MNP products to caregivers^(14,15)^. It also included an evidence-based behaviour change programme to improve infant and young child feeding (IYCF) practices and use of MNP. MNP was delivered by female frontline CHWs called *Shasthya Shebika* (SS) who are volunteers, and by paid workers – *Shasthya Kormi* (SK). The SK supervise the SS who work with the target households at the community level. These SS are the core of BRAC’s community-based health interventions, serving as the first point of contact between community members and BRAC’s health and nutrition services. Each SS is responsible for a range of households depending on their competency, willingness and respective programme modalities. As volunteer CHWs, the SS receive a modest financial incentive for their work, which includes disseminating health and nutrition messages, health screening, providing treatment for common illnesses and selling BRAC’s products (which include MNP, locally branded as Pushtikona-5).

Bangladesh consists of sixty-four districts and 492 sub-districts. The home fortification programme was implemented across a selection of Bangladesh’s 164 sub-districts. In phase one (2014), it was rolled out in sixty-eight sub-districts, selected from ten districts; phase two (2015) started in fifty sub-districts (from fifteen districts); and in phase three (2016), implementation commenced in forty-six sub-districts (from nine districts). This paper is an analysis of phase one data: (1) to quantify the change in coverage achieved from baseline to endline, (2) to ascertain the association between SS’s home visits and measures of MNP coverage and (3) to explore factors associated with MNP coverage.

## Methods

### Interventions

Five key interventions under the MIYCN programme were implemented to improve programme coverage. These were: (i) a basic training course for SS and monthly refresher sessions to promote home fortification with MNP at the household level; (ii) regular home visits by SS who provided advice to caregivers of target children on the programme; (iii) community-level monitoring of, and support to, SS’s home fortification activities; (iv) advocacy about the programme aimed at national-level key stakeholders and community gatekeepers to increase the awareness of MNP; and (v) incentivising SS to improve compliance with the programme at the community level. BRAC provided training to the SS to increase programme coverage, including sale and use of MNP products at the household level based on a training module developed with the support of GAIN and other key stakeholders who are experts in MIYCN interventions. This was implemented by the Training Department of BRAC, with a team of specialist trainers, including experts on home fortification with MNP.

### Data source

This study used data from three coverage surveys to evaluate phase one of the MIYCN programme in Bangladesh using a pre–post study design. Data were collected at three time-points; (i) at baseline prior to implementing the intervention; (ii) at midline, 1 year after baseline and (iii) at endline, 2 years after the midline survey. The baseline, midline and endline surveys were implemented at the same period of the year (September 2014, 2015 and 2017) to control for potential seasonal effects.

### Study population

The study population was children aged 6–59 months and their caregivers. A caregiver was defined as the child’s biological mother or the person who cares for, or looks after and gives the child the most meals on most days. For this study, we considered an eligible household as a dwelling with at least one woman of reproductive age who had a child aged 6–59 months.

### Sampling

**Fig. 1:**
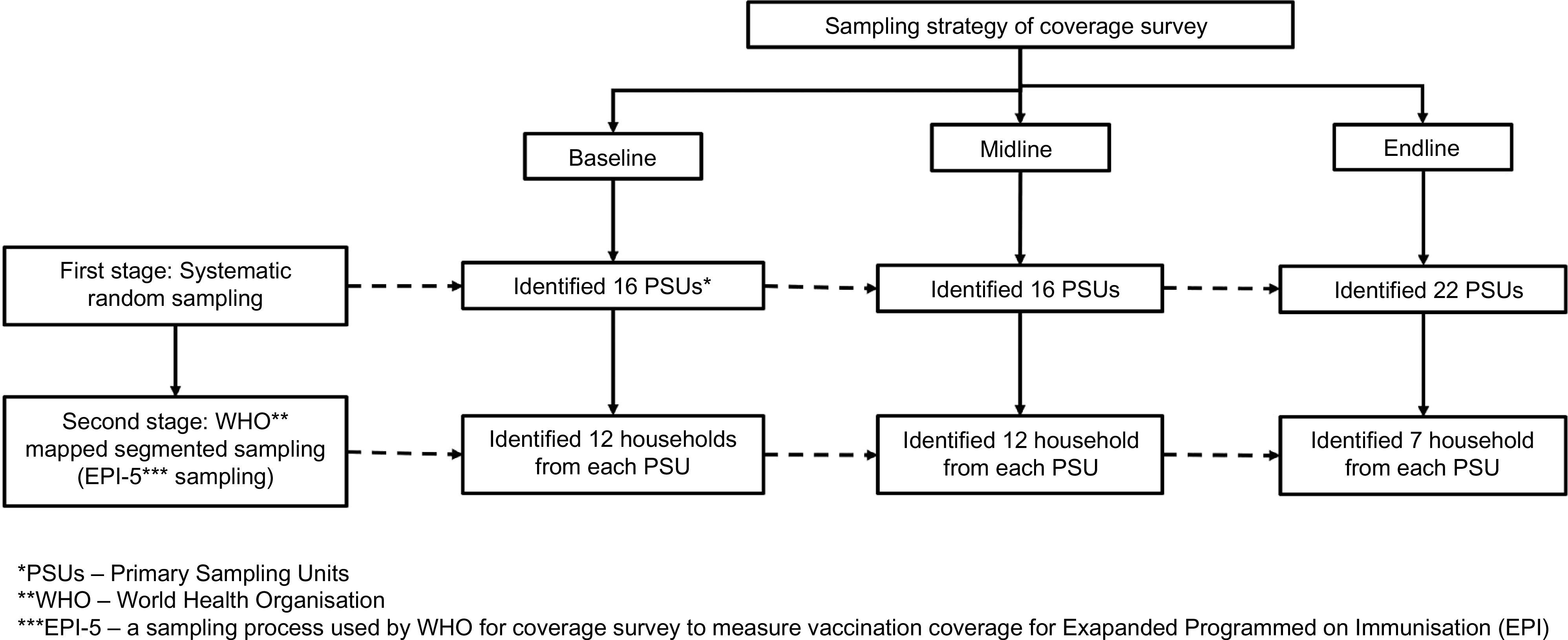
Two-stage sampling strategy for the evaluation of MIYCN home fortification with MNP. PSU, primary sampling units; EPI-5, a sampling process used by the WHO for coverage survey of EPI

A two-stage cluster sampling procedure, stratified by district, was employed to select households for inclusion in the study (Fig. 1). All ten districts included in phase one were included in the sample. In the first stage, systematic random sampling was used to select BRAC communities as primary sampling units (PSUs) with equal probability of selecting any community or PSU from rural sub-districts within each district. We randomly selected the first PSU, then the next PSU was identified using a sampling interval. The sampling interval was calculated by dividing the total number of PSUs in each district by the desired number of PSUs. For the baseline and midline surveys, we selected sixteen PSUs from each district. However, in the endline survey, we sampled twenty-two PSUs to cover more PSUs from each district.

In the second stage of sampling, we identified twelve households from each PSU in the baseline and midline survey and increased the number of PSUs along with an additional seven households from each PSU in the endline survey. To identify households, we followed the WHO’s EPI-5 (Expanded Programmed on Immunisation) sampling procedure^(16)^. On the day of interviewing, the survey team went to the selected PSU and hand-drew a map of the PSU in consultation with local community leaders who identified landmarks, households and other important features. The team divided the PSU into four segments and identified the middle point of each segment. From this point, we spun a bottle to identify a direction and starting point from which to count households. Every fifth household was interviewed if it contained an eligible child aged 5–59 months and a caregiver physically and mentally competent to provide consent. If the eligible household had more than one eligible child and/or caregiver, we randomly selected (by lottery) one child and their caregiver. If the household did not have an eligible child, we searched for the next fifth household to the right. If we did not find an eligible child in three attempts, we spun the bottle from the current place and followed the same procedure until we found an eligible household. Once we had located an eligible household, we used the same procedure to locate the next ones. Once three eligible households had been recruited, another segment was randomly selected and the same procedure was followed. In the endline survey, we recruited two households in the first three segments, and one household from the fourth segment.

### Data collection

Our structured questionnaire covered standard IYCF indicators and other national and international guidelines relating to MIYCN^(17)^. We included four IYCF indicators: continuous breastfeeding, timely initiation of complementary feeding, minimum acceptation diet and minimum dietary diversity. The survey instrument was adapted from the fortification assessment coverage toolkit developed by the Global Alliance for Improved Nutrition^(18)^. We conducted two tests in real field settings in non-survey areas and incorporated the feedback into the final version of the questionnaire.

The final questionnaire included 254 items under sixteen sections: (1) household information; (2) child’s and caregiver’s demographic characteristics; (3) household assets; (4) household food security status; (5) exposure to home fortification with MNP; (6) household water and sanitation facilities; (7) food security status of the household; (8) child’s morbidity; (9) child’s history of taking medicine; (10) IYCF practices; (11) mother’s dietary diversity; (12) household exposure to industry-fortified foods; for example, fortified salt and oil; (13) caregiver exposure to advice from an SS on home fortification with MNP; (14) nutrition status of the mother and child; (15) immunisation coverage; and (16) home visit by BRAC’s SS. We asked caregivers whether the household had ever been visited by a BRAC’s SS. If they said yes, we asked whether the SS visited in the last 12 months (interviewers were trained to help caregivers recall the time when the SS visited them by referring to different events of the year, such as before the last rainy season, last winter or child’s birthday, etc.).

We used electronic data collection procedures for recording survey data. A team from the Information Technology Department of icddr,b provided technical support to develop an Android-based smartphone programme and to design a data entry application based on the survey questionnaire. To support the Android operating system, we used the Open Data Kit (ODK) software for developing the programme. In addition to the survey interview, we collected and recorded GPS data on caregivers’ households in all surveys. Trained interviewers administered the questionnaire to the caregivers. The team leaders independently re-interviewed 5 % of the interviewees randomly to check and ensure the quality and accuracy of data.

### Outcome variables

The coverage of home fortification with MNP is the main outcome of this analysis. The FACT coverage survey methodology^(6)^ was developed based on the Tanahashi model^(8)^, including three types of coverage: message coverage, contact coverage and effective coverage. In this study, we defined message coverage as whether the caregiver had *ever heard* about MNP. Contact coverage was whether the caregiver had *ever given* MNP to the participant child. Finally, effective coverage was defined as how *frequently the caregiver administered* MNP, in accordance with programme recommendation. To measure effective coverage, we asked caregivers whether they provided MNP mixed with food to their children on at least three of the 7 d prior to the survey.

### Exposure variable

Since all of BRAC’s community health and nutrition interventions are delivered by their CHWs, it was assumed that households visited by CHWs were exposed to BRAC’s home fortification programme. We defined the exposure variable as the household that had been visited by a BRAC SS (yes or no) within 12 months before the survey.

### Covariates

Based on literature about the coverage of MNP^(6,7,18–21)^, we identified two levels of covariates for this analysis: individual and household. Covariates at the individual level included age of the participant child in months, sex of the child (male or female), age of the caregiver in years and educational status of the father of participant child (years of education completed). Household-level covariates were household size (number of members), religion, number of children aged 6–59 months living in the household, time of most recent birth (when the last child of the household was born) and relative wealth (wealth index) of the household. Based on the Demographic Health Survey method^(22)^, we calculated a household wealth index to measure a household’s cumulative living standard based on ownership of assets, such as televisions and bicycles, materials used for housing construction, and types of water access and sanitation facilities. We categorised wealth index into tertiles: poor, middle and rich.

### Statistical methods

We used Stata (version 15) to analyse the data. The analyses accounted for the sampling design and incorporated sampling weights to adjust for disproportionate sampling (cluster sampling) and non-response. We compared the household, caregiver and child characteristics first across all three surveys and then within each survey between the households that had and had not been visited by an SS within the past 12 months. We estimated the proportion of message, contact and effective coverage on a population basis in the MIYCN programme area. We report coverage outcomes by SS visit status, with 95 % CI separately for each survey. We performed simple logistic regression to estimate the odds ratio (OR) for the association between each of the coverage indicators as the three outcomes (e.g. message, contact and effective coverage) and SS visit within the past 12 months, survey time-point (baseline, midline and endline) and other covariates described above. We then undertook multiple logistic regressions to examine the association between SS’s home visit within the past 12 months and coverage outcomes, adjusted for other covariates. In these analyses, we included an interaction term for SS’s home visit by survey time-points (baseline, midline and endline) to assess how the association between home contact and coverage changed across the three surveys. This was needed as the SS were active in the community and made home visits prior to the implementation of the home fortification programme. Any associations at midline and endline need to account for baseline associations. *P* values for the interaction term indicated whether the effect of home contact changed over time. From the final models, we report the OR (95 % CI) for the association between home contact and coverage at each of the three surveys as well as an intervention effect, which is the ratio of OR for associations at midline and endline relative to baseline OR. This is an indication of how the home fortification programme has impacted coverage beyond the baseline effect of home contact. We used the *lincom* command in Stata for estimating interaction terms and the *testparm* and *estatgof* commands in Stata to perform Wald tests for the covariates and the Hosmer–Lemeshow goodness-of-fit test of the models.

We estimated sample size considering district-level prevalence of anaemia and MNP coverage. This allowed the research team to provide BRAC with district-level data for the respective programme areas. For baseline and midline surveys, we estimated the sample size required to estimate the coverage. Since limited information was available on the coverage of MNP, we assumed 50 % estimated prevalence, precision of ±10 % (for a 95 % CI), and a design effect of 2; the required sample size was estimated at 192 households for each district. For endline surveys, we estimated the sample size in a pre–post design considering the average prevalence of anaemia, good IYCF practices and effective coverage of MNP in the baseline survey. We considered an effect size of a 10 % decrease for anaemia, 14 % increase for good IYCF practice and 5 % increase for effective coverage; 95 % significance level, 90 % power and design effect of 2. The average prevalence of anaemia yielded the highest sample size, which was estimated at 154 households for each of the ten districts, for a total sample size of approximately 1500 for each survey.

## Results

### Demographic characteristics of study participants

We surveyed 1927 caregivers in baseline, 1924 caregivers in midline and 1540 caregivers in endline surveys. Online Supplemental Table S1 shows the demographic characteristics of study participants across the three surveys, while Table 1 provides a comparison of demographic characteristics of study participants by SS visit within the past 12 months for each survey. As shown in online Supplemental Table S1, most of the characteristics were similar across the three surveys, except for caregiver’s and father’s age and caregiver’s religion. Caregiver’s age and father’s age were the lowest in baseline survey, and the proportion of Muslim households increased across the three surveys; however, the differences were small. The prevalence of home visits by SS before 12 months of the survey was 51 % in baseline, 49 % in midline and 47 % in endline surveys, and there were no significant differences between the prevalence of home visits in three surveys (online Supplemental Table S1). Table 1 shows the characteristics of study participants at each of the three surveys by SS visit within the past 12 months. Child’s age, caregiver’s age, father’s age and the proportion of households with a childbirth within the past 12 months were generally higher for households without an SS visit in the last 12 months, compared to households without a visit.

**Table 1:** Demographic characteristics of study participants by SS visit within the past 12 months of the survey

Variable	Baseline SS visit within the past 12 months							Midline SS visit within the past 12 months							Endline SS visit within the past 12 months						
	Yes (*n* 966; 51 %)			No (*n* 961; 49 %)				Yes (*n* 925; 49 %)			No (*n* 999; 51 %)				Yes (*n* 730; 47 %)			No (*n* 810; 53 %)			
	*n*		%	*n*		%	P*	*n*		%	*n*		%	P*	*n*		%	*n*		%	P*
Household size																					
Mean		5·1			5·2		0·669		5·2			5·0		0·160		5·0			5·0		0·578
sd		1·9			2·1				2·0			1·9				1·8			1·8		
Child’s age (months)																					
Mean		25·2			33·8		<0·001		26·7			33·5		<0·001		27·6			31·6		<0·001
sd		14·3			13·2				14·5			14·7				14·5			14·7		
Caregiver’s age (years)																					
Mean		25·4			26·2		0·056		26·0			27·5		<0·001		26·4			27·2		0·013
sd		5·4			5·7				5·7			6·6				5·7			7·0		
Caregiver’s education (≥5 years of schooling)	678		71·1	630		67·2	0·152	664		73·8	663		67·2	0·011	567		78·0	557		67·9	<0·001
Caregiver’s religion, Muslim	851		84·6	857		85·7	0·621	820		89·5	895		89·6	0·947	657		91·4	767		95·0	0·008
Age of father (years)																					
Mean		31·9			33·1		<0·001		32·4			33·9		<0·001		33·4			33·3		0·769
sd		6·4			7·1				7·0			7·9				6·6			7·0		
Father’s education (≥5 years of schooling)	541		57·4	512		56·6	0·765	548		62·4	559		57·9	0·080	463		61·9	472		56·7	0·050
Number of children in the household aged 6–59 months: one child	805		83·0	850		90·0	<0·001	786		85·3	875		88·3	0·075	642		87·4	725		89·8	0·216
Sex of children, female	452		46·5	455		48·4	0·478	438		47·6	478		49·3	0·534	343		47·5	407		49·8	0·425
Time of most recent birth (≤12 months)	229		32·1	79		6·6	<0·001	220		24·6	124		12·9	<0·001	154		21·2	131		15·0	0·004
Wealth index																					
Poor	354		35·7	300		30·5	0·103	317		33·3	331		32·4	0·818	230		30·0	284		35·7	0·102
Middle	300		27·7	332		30·9		312		33·4	323		32·7		247		32·5	270		32·1	
Rich	312		36·6	329		38·6		296		33·3	345		34·9		253		37·6	256		32·2	

* *P* value from *t* test for continuous variables and *χ*^2^ test for categorical variables.

The mean age of children in households visited by an SS compared to those without an SS visit was 25·2 *v*. 33·8 months, respectively; *P* < 0·001 at baseline; 26·7 *v*. 33·5 months (*P* < 0·001) at midline; and 27·6 *v*. 31·6 months (*P* < 0·001) at endline. The mean age of caregivers at midline was 26·0 years for those visited by an SS compared to 27·5 for those without an SS visit (*P* < 0·001) and, at endline, was 26·4 *v*. 27·2 (*P* = 0·013). Mean age of the father was 31·9 *v*. 33·1 at baseline and 32·4 *v*. 33·9 at midline for those with and without an SS visit in the last 12 months, respectively (*P* < 0·001 for both).

Households in which mothers gave birth within 12 months of the survey received more SS visits compared to households in which no childbirth occurred during that period. Remembering that Bangladesh is predominantly a Muslim country, at endline, 91 % of caregivers in households visited by the SS, and 95 % of caregivers in households that were not visited by the SS, were Muslims. At baseline, 83 % of households receiving an SS visit within the last 12 months and 90 % of households without a visit had one child (*v*. more than one) aged 6–59 months (Table 1).

### Univariate, bivariate and multivariable results of coverage indicators

#### Message coverage

**Fig. 2:**
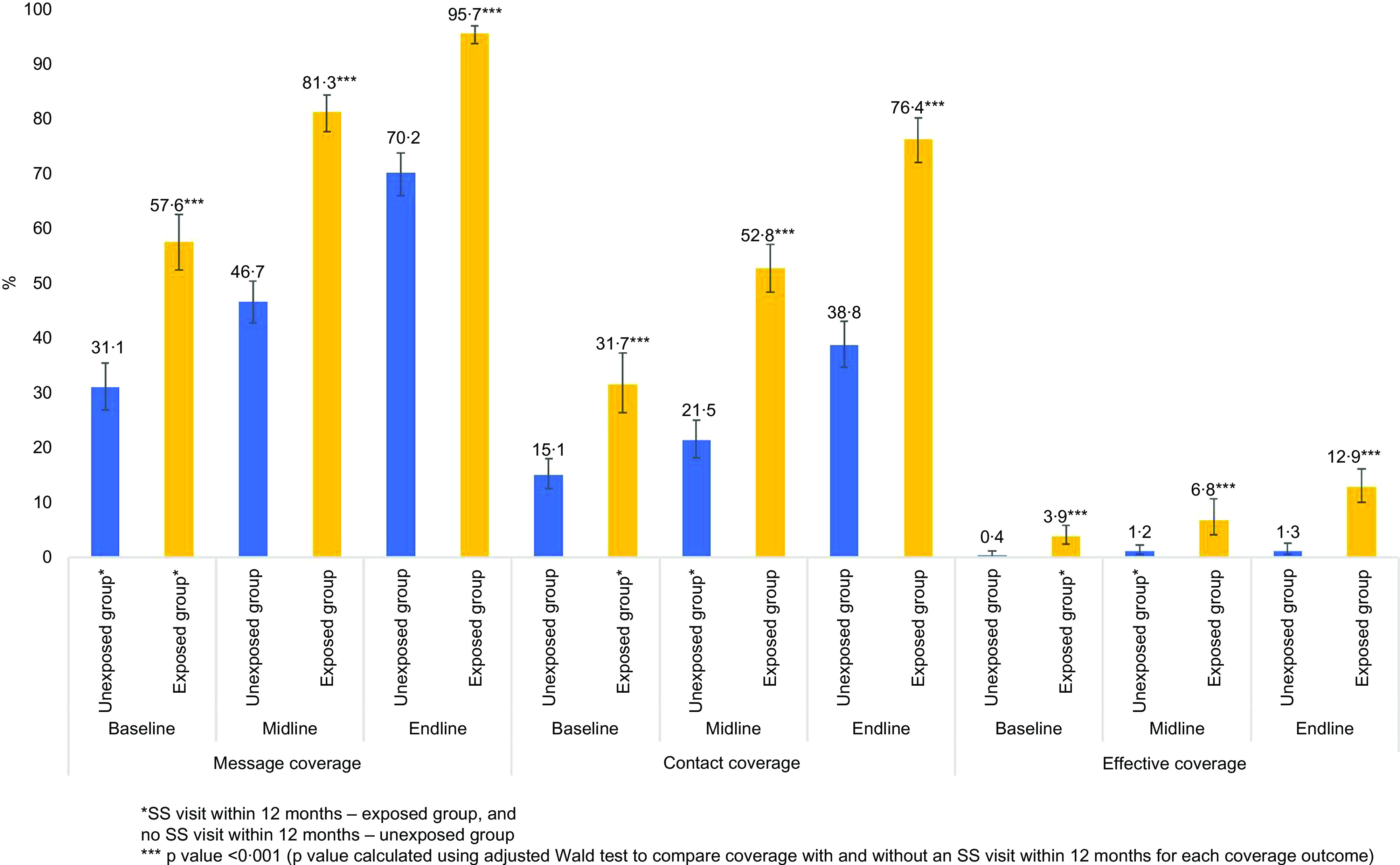
Percentage of households with each coverage indicator in the three surveys by SS visit within the last 12 months of survey.

We observed a significant increase in the proportion of caregivers who had heard about home fortification with MNP from baseline to midline and endline, irrespective of whether they had received an SS visit in the past 12 months (Fig. 2). The proportion of caregivers in the exposed group that received message coverage was 58 % in baseline, 81 % in midline and 96 % in endline (*P* < 0·001). It was 31, 47 and 70 %, respectively, in baseline, midline and endline surveys in the unexposed group (Fig. 2). Bivariate analysis showed that the unadjusted OR of message coverage was significantly higher for households that received at least one visit by the SS before 12 months of the survey compared to households that did not receive any SS visit during the same period (OR 3·46; 95 % CI 2·89, 4·13; *P* < 0·001). Other covariates including child’s age, caregiver’s education, father’s age, father’s education, sex of child, wealth index and survey time points were significantly associated with message coverage of MNP in the bivariate model (Table 2). In the multivariable model, after adjusting for potential confounders, we found that the odds of message coverage was significantly higher in households that had received at least one visit by SS within 12 months of the survey compared to the households not receiving any visit during the same period (adjusted OR (AOR) 2·85; 95 % CI 2·29, 3·56; *P* < 0·001 at baseline; AOR 4·86; 95 % CI 3·90, 6·04; *P* < 0·001 at midline; AOR 10·20; 95 % CI 6·69, 15·57; *P* < 0·001 at endline). The interaction effect demonstrated a statistically significant effect for message coverage at both midline (ratio of OR 1·70; 95 % CI 1·25, 2·32; *P* < 0·01) and endline (ratio of OR 3·58; 95 % CI 2·22, 5·78; *P* < 0·001).

*SS visit within 12 months – exposed group; no SS visit within 12 months – unexposed group.

***P value < 0·001 (*P* value calculated using adjusted Wald test to compare coverage with and without an SS visit within 12 months for each coverage outcome)

**Table 2:** Unadjusted OR of message, contact and effective coverage of MNP with other independent variables

Variable	Message coverage			Contact coverage			Effective coverage		
	OR	95 % CI	P*	OR	95 % CI	*P*	OR	95 % CI	*P*
Household size	0·98	0·94, 1·01	0·192	0·96	0·92, 1·00	0·037	1·01	0·92, 1·11	0·821
Child’s age	0·991	0·988, 0·996	<0·001	1·005	1·002, 1·010	0·007	0·95	0·94, 0·97	<0·001
Caregiver’s age	0·97	0·96, 0·98	<0·001	0·98	0·97, 0·99	<0·001	0·97	0·94, 1·00	0·029
Caregiver’s education (Ref: <5 years)	1			1			1		
≥5 years	1·73	1·47, 2·03	<0·001	1·43	1·23, 1·67	<0·001	2·13	1·38, 3·31	0·001
Religion (Ref: Hindu/other)	1			1			1		
Muslim	1·04	0·73, 1·48	0·826	1·18	0·82, 1·70	0·374	0·97	0·56, 1·66	0·903
Father’s age	0·98	0·97, 0·99	<0·001	0·98	0·97, 0·99	0·002	0·98	0·96, 1·01	0·257
Father’s education (Ref: <5 years)	1			1			1		
≥5 years	1·43	1·21, 1·69	<0·001	1·41	1·22, 1·64	<0·001	1·51	1·08, 2·10	0·015
Sex of child (Ref: male)	1			1			1		
Female	0·86	0·76, 0·98	0·019	0·85	0·74, 0·98	0·027	0·74	0·55, 1·01	0·056
Number of children aged 6–59 months in the household (Ref. 1)	1			1			1		
≥2	1·23	1·00, 1·53	0·054	0·91	0·74, 1·11	0·346	1·19	0·77, 1·85	0·437
Time of most recent birth (Ref: >12 months)	1			1			1		
≤12 months	1·01	0·84, 1·21	0·913	0·62	0·50, 0·77	<0·001	2·64	1·76, 3·95	<0·001
Wealth index (Ref: poor)	1			1			1		
Middle	1·24	1·05, 1·46	0·012	1·10	0·93, 1·30	0·248	1·39	0·89, 2·18	0·152
Rich	1·41	1·16, 1·71	0·001	1·20	0·99, 1·46	0·068	1·55	1·01, 2·38	0·046
Survey time-points (Ref: baseline)	1			1			1		
Midline	2·18	1·78, 2·68	<0·001	1·90	1·52, 2·37	<0·001	1·89	1·03, 3·40	0·041
Endline	5·77	4·63, 7·19	<0·001	4·24	3·44, 5·23	<0·001	3·03	2·07, 5·26	<0·001
Home visit by SS within 12 months (Ref: no visit)	1			1			1		
Yes	3·46	2·89, 4·13	<0·001	3·24	2·73, 3·85	<0·001	8·13	4·78, 13·83	<0·001

* *P* values <0·05 were regarded as statistically significant.

#### Contact coverage

An increasing trend was observed for contact coverage, where the caregiver gave MNP to the target child (32 % at baseline, 53 % at midline, 76 % at endline in the group with SS visit within the past 12 months, *P* < 0·001), whereas, in the unexposed group, contact coverage was 15, 22 and 39 % at baseline, midline and endline surveys, respectively (Fig. 1). In the bivariate model, contact coverage was significantly associated with an SS visit within 12 months to the household (OR 3·24; 95 % CI 2·73, 3·85; *P* < 0·001) (Table 2). Household size, child’s age, caregiver’s age, caregiver’s education, father’s age, father’s education, child’s sex, most recent birth in the household, wealth index and survey time points were also significantly associated with contact coverage of MNP. In the adjusted model, we found that the odds of coverage was significantly higher in the households that received at least one visit by the SS before 12 months of the survey compared to households not receiving any visit during the same period (AOR 3·35; 95 % CI 2·61, 4·31; *P* < 0·001 at baseline; AOR 4·98; 95 % CI 3·97, 6·22; *P* < 0·001 at midline; AOR 5·84, 95 % CI 4·58, 7·45; *P* < 0·001 at endline surveys). The interaction effect demonstrates a statistically significant effect for contact coverage at both midline (ratio of OR 1·48; 95 % CI 1·06, 2·07; *P* < 0·05) and endline (ratio of OR 1·74; 95 % CI 1·23, 2·47; *P* < 0·01) (Table 3).

**Table 3:** Adjusted OR (AOR) of message, contact and effective coverage of MNP with other independent variables (multivariable regression model) and the use of interaction terms for home contact of SS *v*. survey time-points

Variable	Message coverage			Contact coverage			Effective coverage		
	AOR	95 % CI	P*	AOR	95 % CI	*P*	AOR	95 % CI	*P*
Household size	0·96	0·91, 0·99	0·014	0·97	0·94, 1·01	0·098	0·94	0·87, 1·03	0·186
Child’s age	1·00	0·99, 1·002	0·188	1·01	1·01, 1·02	<0·001	0·97	0·96, 0·99	<0·001
Caregiver’s age	0·98	0·96, 0·99	0·001	0·98	0·97, 0·99	0·027	1·00	0·96, 1·05	0·854
Caregiver’s education (Ref: <5 years)	1			1			1		
≥5 years	1·31	1·12, 1·54	0·001	1·11	0·94, 1·31	0·215	1·41	0·90, 2·19	0·132
Religion (Ref: Hindu/other)	1			1			1		
Muslim	0·85	0·65, 1·11	0·225	0·89	0·69, 1·13	0·330	0·59	0·37, 0·94	0·923
Father’s age	0·996	0·98, 1·01	0·545	0·99	0·98, 1·01	0·295	1·01	0·98, 1·04	0·026
Father’s education (Ref: <5 years)	1			1			1		
≥5 years	1·12	0·97, 1·30	0·116	1·16	1·00, 1·34	0·048	0·98	0·72, 1·33	0·876
Sex of child (Ref: male)	1			1			1		
Female	0·83	0·74, 0·94	0·003	0·83	0·73, 0·94	0·003	0·81	0·62, 1·06	0·121
Number of children in the household aged 6– months (Ref. 1)	1			1			1		
≥2	1·38	1·13, 1·69	0·002	0·99	0·82, 1·20	0·929	1·05	0·69, 1·59	0·830
Time of most recent birth (Ref: >12 months)	1			1			1		
≤12 months	0·61	0·50, 0·74	<0·001	0·44	0·36, 0·54	<0·001	1·34	0·90, 2·01	0·152
Wealth index (Ref: poor)	1			1			1		
Middle	1·20	1·03, 1·41	0·021	1·10	0·94, 1·29	0·251	1·29	0·87, 1·91	0·205
Rich	1·38	1·17, 1·64	<0·001	1·20	1·01, 1·42	0·039	1·53	1·02, 2·29	0·039
Association between home visit and coverage at each time-point									
SS visit *v.* no SS visit – baseline	2·85	2·29, 3·56	<0·001	3·35	2·61, 4·31	<0·001	9·34	2·83, 31·09	<0·001
SS visit *v.* no SS visit – midline	4·86	3·90, 6·04	<0·001	4·98	3·97, 6·22	<0·001	5·40	2·39, 12·21	<0·001
SS visit *v.* no SS visit – endline	10·20	6·69, 15·57	<0·001	5·84	4·58, 7·45	<0·001	12·68	6·09, 26·44	<0·001
Intervention effect†									
Ratio of midline OR to baseline OR	1·70	1·25, 2·32	0·001	1·48	1·06, 2·07	0·021	0·58	0·14, 2·44	0·455
Ratio of endline OR to baseline OR	3·58	2·22, 5·78	<0·001	1·74	1·23, 2·47	0·002	1·35	0·33, 5·48	0·673

* *Z*-test (*P* values <0·05 were regarded as statistically significant).

† Intervention effect is the OR for the association between home visit and coverage at midline or endline, divided by the OR for the association between home visit and coverage at baseline; this measure – which is not a true OR, rather a ‘ratio of ORs’ – estimates the association at midline or endline relative to (or ‘adjusted for’) that at baseline.

#### Effective coverage

Effective coverage, or how frequently the caregiver administered MNP, also increased significantly from baseline (4 %) to midline (7 %) and to endline survey (13 %) in the households that received an SS visit before 12 months of the survey (Fig. 1). The bivariate model suggested a significant association between SS home visit and effective coverage (OR 8·13; 95 % CI 4·78, 13·83; *P* < 0·001) compared to households that did not receive an SS visit (Table 2). This analysis also indicates that fathers who had ≥5 years of schooling compared to those with <5 years of schooling, rich households compared to poor ones in terms of the wealth index, and households in endline survey compared to baseline were significantly associated with increased effective coverage. In the adjusted model, we did not observe any significant association between SS home visits and effective coverage (in the interaction term, the ratio of endline OR to baseline OR 1·35; 95 % CI 0·33, 5·48; *P* = 0·673) (Table 3).

## Discussion

Home visits by BRAC’s CHWs are crucial to improving the programme coverage. Our analysis has demonstrated that caregivers who received visits from the BRAC’s CHWs before 1 year of the survey had received information about the MNP product and its provision to children in their everyday foods. We did not find a similar study in the literature although there is evidence that home visits by CHWs are an effective intervention to improve infants’ and young children’s health^(23,24)^ and maternal health^(25)^. Previous literature has also demonstrated that trained CHWs can help promote exclusive breastfeeding of children until 6 months of age in a developing country setting^(26)^.

An important finding is that contact coverage has consistently increased from baseline to endline, indicating that home visits by CHWs can increase programme coverage. While the proportion of households receiving a SS visit within the past 12 months was similar for the three surveys, all three coverage indicators increased over time. When we looked at the association between an SS visit and coverage across time, using our interaction terms, there was a statistically significant effect for message coverage and contact coverage at both midline and endline surveys. It appears that the home fortification programme was successful in improving coverage over time through SS visits.

Home visits by CHWs helped caregivers gain an understanding about the use of the MNP product for their children. Previous literature suggested that home visits of CHWs have a number of other benefits^(24)^. During home visits, CHWs may provide advice on ideal ways of using the MNP product and other good childcare practices to caregivers^(27,28)^. They do this by interacting with caregivers and discussing their problems with the programme team^(10)^. There are some barriers to a successful implementation of MNP interventions, such as misconceptions around MNP, side-effects of MNP and inconsistent supply of MNP product^(12)^, and regular home visits (expected at least one visit per month) by CHWs might help overcome these^(11,29,30)^.

In addition to the importance of home visits by CHWs, we found that children’s and caregivers’ characteristics influenced coverage. A child’s age is an important factor as better effective coverage was observed among younger children. It is important for younger children to receive effective coverage, as they are at a higher risk of nutritional deficiencies and have the highest micronutrient needs relative to their stage of growth and development. That this age group had a higher effective coverage was likely due to behaviour change communication and IYCF counselling provided by the CHWs. It is probably the reason that most MNP interventions traditionally targeted the children aged 6–23 months compared to older children^(7)^.

Our findings have important policy implications – both at the global and country levels. Globally, there is limited evidence concerning home fortification of foods with MNP at the national level with optimum programme coverage. The few MNP programmes that monitored programme coverage have sometimes provided MNP free rather than using a market-based approach, where CHWs sell the MNP product to caregivers^(14,15)^. The Bangladesh MIYCN home fortification programme may be one of the few programmes in which CHWs sell MNP to caregivers and provide practical advice. This has demonstrated success in achieving message coverage and contact coverage.

A market-based MNP programme may be more financially sustainable compared to a free distribution model^(17,31)^. However, the public health impact of a market-based MNP programme remains unclear, and previous literature has raised concerns around the affordability of MNP by poor communities and indicated that most private sector companies have maximised profit through this model^(31,32)^. Therefore, an appropriate government body is needed to monitor and regulate market-based MNP promotion at the population level. However, alternative sources of bioavailable micronutrient (e.g. iron and zinc)-dense foods are red and organ meat from animals, and these are expensive compared to MNP. For example, in Bangladesh, the price of 1 kg of red meat is BDT 400–500 (BDT 1 = US$0·012), whereas the cost of thirty MNP sachets is BDT 70. Existing literature also shows the challenges in achieving ideal programme coverage^(14)^ due to poor stocking of the MNP product at the community level, insufficient capacity for monitoring the programme, inadequate funding, poor delivery strategies and inadequate training and capacity building for health workers^(33,34)^.

There has been a tradition in Bangladesh of using CHWs to implement a number of high-quality health interventions since the early 1990s, including the EPI programme and a family planning programme^(35)^. EPI in Bangladesh has been implemented under the Directorate General of Health Services (DGHS) of the Ministry of Health and Family Welfare (MOHFWA). The DGHS has dedicated CHWs, called health assistants, operating at the community level; they are responsible for implementing immunisation programmes in Bangladesh. Similarly, female family welfare assistants are involved in implementing family planning interventions under the Directorate General of Family Planning of MOHFW. Health assistants and family welfare assistants of the MOHFW are paid health workers who are responsible for implementing targeted interventions (e.g. EPI and family planning). However, there are no CHWs available at the MOHFW particularly dedicated to implementing nutrition interventions in Bangladesh. In such situations, the implementation of nutrition interventions at the community level using CHWs working with different NGOs is crucial. BRAC’s CHWs have demonstrated their abilities to implement nutrition interventions with improved programme coverage, showing a greater potentiality for collaboration towards filling the human resource gaps within the MOHFW in Bangladesh.

The successful implementation of the MIYCN programme using BRAC’s CHWs has demonstrated how the MOHFW could explore and utilise the diverse sources of human resources for improving the health and nutrition of the country’s population. There is a potential for the MOHFW to collaborate with BRAC’s CHWs to implement nutrition interventions targeted in the current Operation Plan (OP) of the National Nutrition Services. During the previous OP (2006–11), the country had struggled to implement the full spectrum of its interventions^(19)^.

### Limitations

The MIYCN programme has been implemented at the national level. We used a concurrent evaluation, which may not be a gold standard because we did not have a randomised comparison group. This limited our ability to control other factors that might have impacted the coverage of MNP. In order to address this limitation, we employed several analytic strategies, including the use of an interaction term, which allowed us to conclude that there were changes in the association between SS visits and coverage over time. Our exposure variable – at least one SS home visit within 12 months – may not be sensitive enough to assess all coverage indicators. We did not have data on the frequency of home visits, which limited a further analysis of effective coverage, although previous qualitative results showed that regular SS home visits encouraged mothers to regularly use MNP^(12)^. Results of this study may not be representative of the whole of Bangladesh or for the districts that implemented the surveys. There was limited information available to identify the actual location and size of the communities in which the BRAC operated, and the selected houses might overlap with catchment areas of CHWs employed by other organisations.

## Conclusion

Households visited by BRAC’s volunteer CHWs had better message and contact coverage, although the overall effective coverage was very low. The study suggests that if BRAC’s SS are given more training and programmatic support, they can play a vital role in increasing the coverage of MNP programmes in Bangladesh. BRAC may need additional SS included in its CHW-based service delivery networks to ensure frequent home visits to improve effective coverage. Based on these findings, we recommend scaling up the MIYCN intervention further to improve MNP coverage in similar settings where children suffer from high levels of micronutrient deficiencies. However, given the design of this evaluation, it is important to interpret our conclusions cautiously.

## Supplementary material

For supplementary material accompanying this paper visit https://doi.org/10.1017/S1368980020000038. click here to view supplementary material
