# Supplementary material for: Role of home visits by volunteer community health workers: to improve the coverage of micronutrient powders in rural Bangladesh
Source: Public Health Nutr. 2020 Mar 5;24(Suppl 1):s48–58. doi: 10.1017/S1368980020000038 (PMC8042577; doi:10.1017/S1368980020000038)
Supplement: Supplementary file 1 [file S1368980020000038sup001.docx]

**Additional analysis**

**Supplementary Table 1.** Background characteristics of study participants at baseline, midline and endline surveys

| **Variable** | **Baseline**  **(n=1927)** | **Midline**  **(n=1924)** | **Endline (n=1540)** | **p-value** |
| --- | --- | --- | --- | --- |
| Household-size, Mean (SD) | 5.1 (2.0) | 5.1 (1.9) | 5.0 (1.8) | 0.580^¶^ |
| Child’s age (in months), Mean (SD) | 29.5 (14.4) | 30.2 (15.0) | 29.7 (14.8) | 0.138^¶^ |
| Caregiver’s age (in years) Mean (SD) | 25.8 (5.5) | 26.7 (6.2) | 26.8 (6.5) | <0.001^¶^ |
| Caregiver’s education (≥5 years schooling), n (%) | 1308 (69) | 1327 (71) | 1124 (73) | 0.295 |
| Caregiver’s religion, Muslim, n (%) | 1708 (86) | 1715 (90) | 1424 (93) | 0.031 |
| Age (years) of father, mean (SD) | 32.5 (6.8) | 33.2 (7.5) | 33.3 (6.8) | 0.001^¶^ |
| Father’s education, (≥5 years schooling), n (%) | 1053 (57) | 1107 (60) | 935 (59) | 0.399 |
| Number of children in the household aged 6-59 months 6-59 months: One child, n(%) | 1655 (87) | 1661 (87) | 1367(89) | 0.258 |
| Sex of children, Female, n(%) | 1020 (53) | 1008 (52) | 790 (51) | 0.755 |
| Time of most recent birth, ≤ 12 Months, n(%) | 378 (20) | 344 (19) | 285 (18) | 0.653 |
| Wealth index, Poor, n(%) | 654 (33) | 648 (33) | 514 (33) | 0.415 |
| Middle, n(%) | 632 (29) | 635 (33) | 517 (32) |  |
| Rich, n(%) | 641 (38) | 641 (34) | 509 (35) |  |
| Home-visit of SS within 12 months of survey,yes, n(%) | 966 (51) | 925 (49) | 730 (47) | 0.506 |

^¶^ p-value from ANOVA for continuous variables (e.g. household-size, child’s age, caregiver’s age, and father’s age)

and chi-square test for categorical variables
